# Specific Antibody Fragment Ligand Traps Blocking FGF1 Activity

**DOI:** 10.3390/ijms19092470

**Published:** 2018-08-21

**Authors:** Julia Chudzian, Anna Szlachcic, Malgorzata Zakrzewska, Miroslawa Czub, Marcin Pustula, Tad A. Holak, Jacek Otlewski

**Affiliations:** 1 Department of Protein Engineering, Faculty of Biotechnology, University of Wroclaw, Joliot-Curie 14A, 50-383 Wroclaw, Poland; julia.chudzian@uwr.edu.pl (J.C.); anna.szlachcic@uwr.edu.pl (A.S.); malgorzata.zakrzewska@uwr.edu.pl (M.Z.); 2 Faculty of Chemistry, Jagiellonian University, Gronostajowa 2, 30-387 Krakow, Poland; miroslawa.luty@gmail.com (M.C.); pustula.marcin@gmail.com (M.P.); holak@chemia.uj.edu.pl (T.A.H.)

**Keywords:** scFv antibody fragments, scFv-Fc fusions, phage display, FGF1, FGFR, ligand trap, tumor targeting treatment, growth factor receptor targeting

## Abstract

Fibroblast growth factor 1 (FGF1) and its receptors (FGFRs) regulate crucial biological processes such as cell proliferation and differentiation. Aberrant activation of FGFRs by their ligands can promote tumor growth and angiogenesis in many tumor types, including lung or breast cancer. The development of FGF1-targeting molecules with potential implications for the therapy of FGF1-driven tumors is recently being considered a promising approach in the treatment of cancer. In this study we have used phage display selection to find scFv antibody fragments selectively binding FGF1 and preventing it from binding to its receptor. Three identified scFv clones were expressed and characterized with regard to their binding to FGF1 and ability to interfere with FGF1-induced signaling cascades activation. In the next step the scFvs were cloned to scFv-Fc format, as dimeric Fc fusions prove beneficial in prospective therapeutic application. As expected, scFvs-Fc exhibited significantly increased affinity towards FGF1. We observed strong antiproliferative activity of the scFvs and scFvs-Fc in the in vitro cell models. Presented antibody fragments serve as novel FGF1 inhibitors and can be further utilized as powerful tools to use in the studies on the selective cancer therapy.

## 1. Introduction

Deregulated growth factor’s receptors play a fundamental role in the development and progression of a variety of human cancers, and are one of the intensively studied therapeutic targets. We focused on fibroblast growth factor 1 (FGF1) and its receptor (FGFR), a member of receptor tyrosine kinase family (RTKs), as its overexpression or aberrant activation has been reported in breast [1,2], lung [3], and gastric cancers [4]. Moreover, the importance of FGFR-targeted therapy lies also in the interplay and complementarity between different growth factor receptors and their downstream signaling—it has been reported that for EGFR, VEGFR, and FGFR, inhibition of one of them leads to compensation in signaling from the remaining ones [5,6]. Additionally, angiogenic properties of FGFRs may further facilitate cancer progression and tumor growth.

Based on the role of the FGF1–FGFR signaling axis in cancer, a number of novel drugs targeting this pathway have been developed and are recently undergoing preclinical and clinical trials in various FGFR-related tumors [7,8]. Current FGFR activation inhibitors can be divided into three groups: small molecule receptor tyrosine kinase inhibitors (TKIs), antagonistic antibody or peptide inhibitors, and FGF ligand traps. TKIs suffer from relatively low specificity towards individual growth factor receptors and thus present a risk of increased side-effects when administered clinically [9].

FGFR-targeted antibodies and peptide inhibitors act by binding to the extracellular domain of the receptor and competing for binding with its ligand, a strategy that proved to be effective i.e., in the clinical use of trastuzumab blocking HER2 receptor [10]. Several antibodies targeting different members of FGFR family have been developed [11]. For example, anti-FGFR2 antibodies displayed high efficacy in mice against FGFR-overexpressing breast cancer (GP369 and GAL-FR22) and gastric cancer (GAL-FR21) xenografts [12,13,14]. MFGR1877S, an anti-FGFR3 antibody, showed very promising results in preclinical studies, but tested in phase I clinical study for urothelial cancers and multiple myeloma yielded equivocal results [15].

The last group of molecules disrupting FGFR signaling are ligand traps that sequester growth factor ligand precluding its binding to the receptor, and thereby inhibiting FGFR activation. Ligand traps are based either on FGFR fragments acting as decoy molecules [16,17], peptides derived from natural FGF binders [18,19], or anti-FGF antibodies. Currently known anti-FGF antibodies have been developed mostly against FGF2, either generated in mice [20] or selected by screening libraries of formats such as scFv [21] and later reformatted to diabody [22]. Besides FGF2, mouse antibodies have been generated also against FGF1, FGF8b, and FGF23 and were shown to inhibit growth of breast and prostate cancer cells [23,24,25].

With fibroblast growth factors and their receptors being increasingly studied and taking into account their role in cancer-directed therapy, in this study we report the development of specific human antibody fragments, targeting fibroblast growth factor 1, that act as FGF1 ligand traps. The binders in scFv format were selected from Tomlinson I and J libraries, fully characterized, and reformatted to bivalent scFv-Fc fusions. Their effectiveness was evaluated by detailed binding tests, as well as investigating their ability to inhibit FGF1-induced cell proliferation. Our results indicate that developed scFvs and scFvs-Fc reduce the in vitro proliferation of FGFR1-expressing mouse fibroblast NIH/3T3cells, FGFR1-transfected BaF3 cells, and most importantly G-292 human osteosarcoma cancer cells.

## 2. Results

### 2.1. Selection of Human scFv Antibody Fragments Specific for FGF1

**Figure 1:**
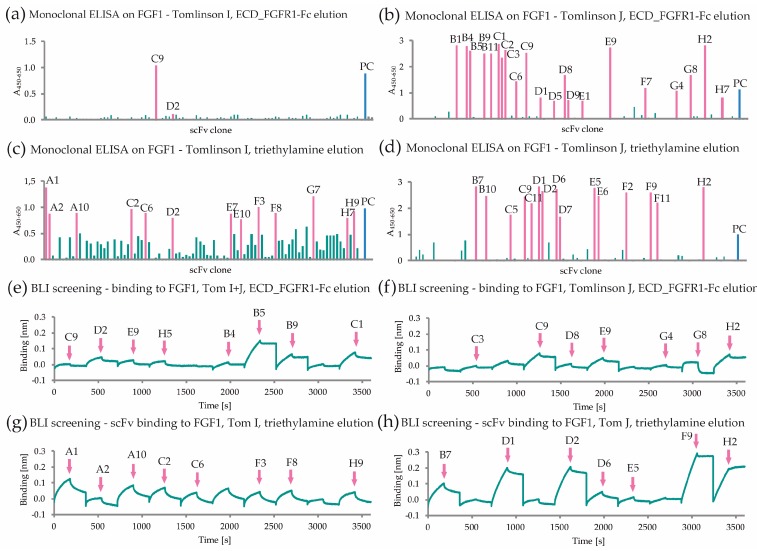
Analysis of anti-FGF1 scFv clones selected from Tomlinson I and J libraries. (**a**,**b**) ELISA signal (green) for the clones eluted using ECD_FGFR1-Fc and (**c**,**d**) triethylamine. Clones giving the highest signal (marked in pink) were selected for further analysis. Anti-BSA clone (PC, indicated in blue) served as a positive control. Grey bars represent negative controls; and (**e**–**h**) BLI screening of scFv clones bacterial supernatants against FGF1 immobilized on the sensor surface (binding signal shown in green). Clones with the best binding profiles (indicated with pink arrow) were chosen for further testing. All scFv names originate from the 96-well plate location of the picked clone.

FGF1-specific scFv antibody fragments were selected from the Tomlinson I + J libraries. In addition to the actual selection of scFvs binding FGF1, each round of selection included counterselection with FGF1 mutant characterized by dramatically reduced affinity to FGFR (FGF1 Y94A/N95A) [26]. The use of this protein in the counterselection step enabled significant depletion of phage clones presenting scFv fragments recognizing receptor-binding deficient FGF1 variant and increased the probability that clones selected afterwards against wild-type FGF1 will bind to the region involved in the interaction with the receptor. In the third round of selection additional competitive elution performed with ECD_FGFR1-Fc was used to obtain a pool of phages presenting scFvs more likely to be specific for the receptor interaction region on FGF1. After three rounds of panning, bacterial supernatants of 368 randomly picked clones were screened for FGF1 binding and 51 FGF1-specific clones were identified based on signal intensity in ELISA assay with immobilized FGF1 (Figure 1a–d). Next, the clones were further validated for binding to FGF1 with the use of biolayer interferometry (BLI). Among tested clones, 30 showed favorable binding profiles (Figure 1e–h) and their nucleotide sequences were analyzed, revealing that several clones shared the same sequence. The overall analysis enabled us to distinguish 10 unique amino acid sequences of FGF1-binding scFvs.

### 2.2. Purification of the Generated FGF1-Specific scFvs

**Figure 2:**
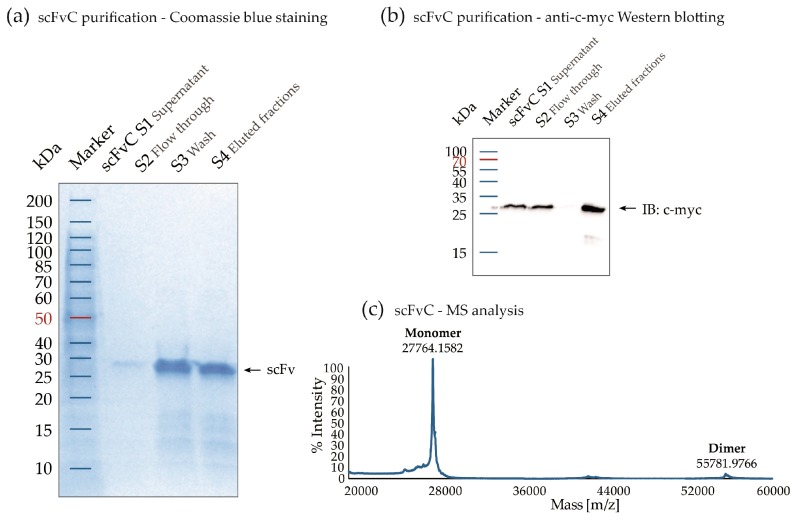
scFv protein expression and purification at an example of scFvC. (**a**) Coomassie brilliant blue staining after SDS-PAGE analysis; (**b**) representative immunoblotting (IB) analysis of scFv purification process. The membrane was stained with anti-c-myc antibodies and proper HRP-conjugated secondary antibodies; and (**c**) the exemplary mass spectrometry spectrum with the highest peak originating from the monomeric fraction of the scFv with the expected molecular mass. Minor peak corresponding to the molecular weight of the covalent dimer was also detected.

We have chosen three scFv variants (named: scFvA, scFvC, and scFvD), based on their FGF1-binding properties and distinct amino acid sequences in the CDR2 and CDR3 loops of the heavy and light antibody chains, that are particularly important in making contacts with the antigen. The scFvs were expressed in HB2151 *E. coli* cells, and efficiency of their production varied from 3.2 to 29.7 mg from one liter of bacterial culture (Table S1). The proteins were purified using Protein A affinity chromatography. Purified scFv fragments were analyzed by gel electrophoresis under reducing conditions, followed by Coomassie brilliant blue staining and anti-c-myc Western blotting (Figure 2a,b). The calculated molecular weights of the recombinant proteins were confirmed using MALDI-MS analysis (Figure 2c). All scFv fragments showed similar purification results.

### 2.3. Characterization, Analysis of Binding to FGF1 and Kinetic Parameters of the Selected scFvs

**Figure 3:**
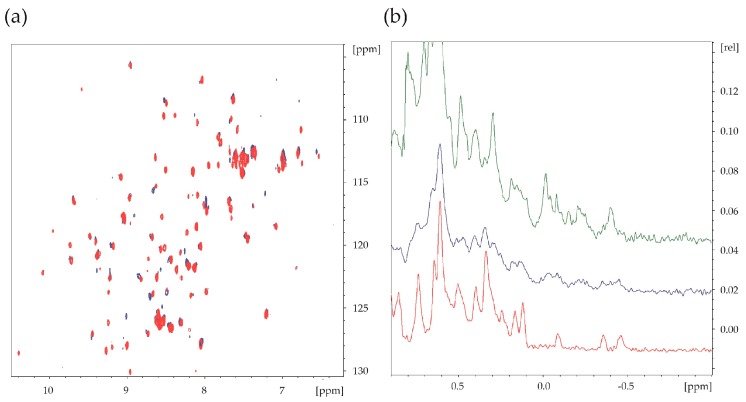
NMR binding assay. (**a**) Exemplary ^15^N-^1^H HMQC NMR spectra of ^15^N FGF1 (red) and the ^15^N FGF1-scFvA complex (blue) (the protein/ligand ratio 1:1); and (**b**) representative 1D ^1^H NMR spectra of FGF1 (red), scFvA (green), and the FGF1-scFvA complex (blue) (the protein/ligand ratio 1:1).

Binding of the antibody fragments (scFvs A, C, and D) to the FGF1 protein was analyzed by the SAR-by-NMR method using the ^15^N labeled FGF1. In all recorded NMR spectra the chemical shifts of a high number of FGF1 NH signals were perturbed upon titration of ^15^N FGF1 with the scFvs (Figure 3a). scFv-induced changes were mostly detected by the broadening of the NMR signals and reduction of signal intensities, indicating complex formation. Quantification of scFvs binding to FGF1 was not possible, since a large number of FGF1 NH signals disappeared upon addition of scFv, with complete lack of signal in the HMQC spectra with higher than equimolar amounts of scFv. Formed FGF1-scFv complex is approximately twice the molecular weight of free FGF1, and thus extensive peak broadening leads to loss of signals. The HMQC NMR results are further supported by the ^1^H NMR spectra (Figure 3b).

**Figure 4:**
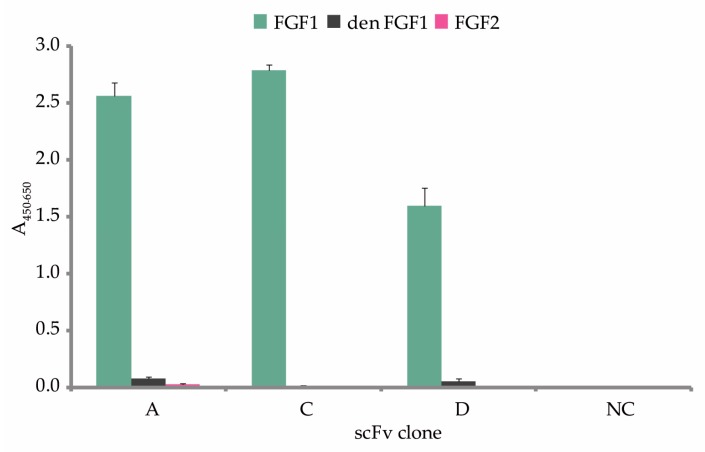
ELISA cross-reactivity analysis. Purified scFv fragments were tested for specificity against native FGF1 with thermally denatured FGF1 (den FGF1) and FGF2 serving as negative controls. A non-FGF1-specific scFv clone was used as an additional negative control (NC). The error bars show standard deviation.

To test if the generated scFvs bind specifically to properly folded FGF1, we performed ELISA assay with immobilized native FGF1. Thermally denatured FGF1 and native FGF2 were used as controls for scFv binding specificity (Figure 4). We confirmed that all three antibody fragments (scFvs A, C, and D) recognized native FGF1, without binding structurally similar FGF2.

**Figure 5:**
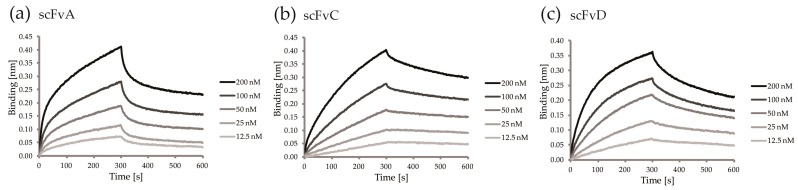
Binding profiles of the selected scFv fragments to FGF1. (**a**–**c**) Serial twofold dilutions of the scFvs were analyzed for interaction with chemically immobilized FGF1, and dissociation measured in kinetics buffer (KB).

The affinity to FGF1 and the parameters of the analyzed scFvs were determined with BLI kinetic measurements. We observed strong binding of the antibody fragments to FGF1 chemically immobilized on the sensor (Figure 5a–c). The data were fitted using a bivalent analyte interaction model, due to the fact that the antibody fragments can partly associate to form a mixture of dimers and monomers. The calculation of the kinetic parameters revealed that the selected scFvs displayed high binding affinities with K_D_ values in the nano- and micromolar range, as summarized in Table 1.

**Figure 6:**
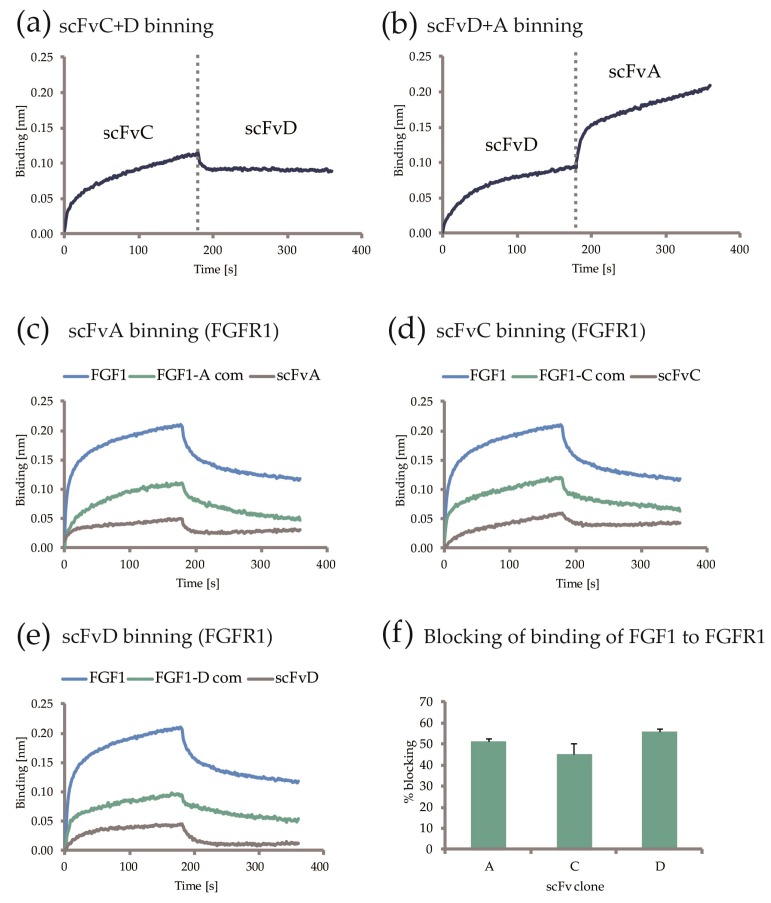
Epitope binning of selected scFvs with FGF1 and ECD_FGFR1-Fc. (**a**,**b**) Representative sensograms showing binding of subsequent scFvs to FGF1 immobilized on the sensor. scFvC blocking the ability of scFvD to bind FGF1, and scFvA binding FGF1 in the presence of scFvD; (**c**–**e**) Binding of FGF1 (blue), FGF1-scFv complex (green), and scFv (grey) to ECD_FGFR1-Fc immobilized on the sensor; (**f**) Percent of blocking of FGF1 binding to ECD_FGFR1-Fc by the selected antibody fragments. The error bars indicate standard deviation.

To verify if the regions within FGF1 to which selected monoclonal antibody fragments bind are distinct or overlapping, epitope binning was performed with the use of BLI method. In a series of BLI measurements, pairs of scFvs were tested for binding to chemically immobilized FGF1. Subsequent incubation of FGF1 with different scFv was used to verify if scFvs were able to bind FGF1 simultaneously at distinct epitopes, or if the first scFv precluded the second scFv from binding to the ligand (Figure 6a,b). As a result of the study two independent binding epitopes on FGF1 were distinguished. We found that among three tested scFvs, two of them: scFvC and scFvD share a binding site on FGF1 and compete in binding it, and scFvA binds non-overlapping epitopes on the ligand. In the next step we verified if the scFvs were able to compete with FGFR1 for FGF1 binding. For this approach we used immobilized recombinant ECD_FGFR1-Fc protein and analyzed binding of premixed scFvs-FGF1 complexes, with scFvs or FGF1 only, serving as controls. The experiments revealed that the generated antibody fragments decrease the ability of FGF1 to bind to its receptor (Figure 6c–f).

### 2.4. Reformatting of the scFv Antibody Fragments to the scFv-Fc Fusions

**Figure 7:**
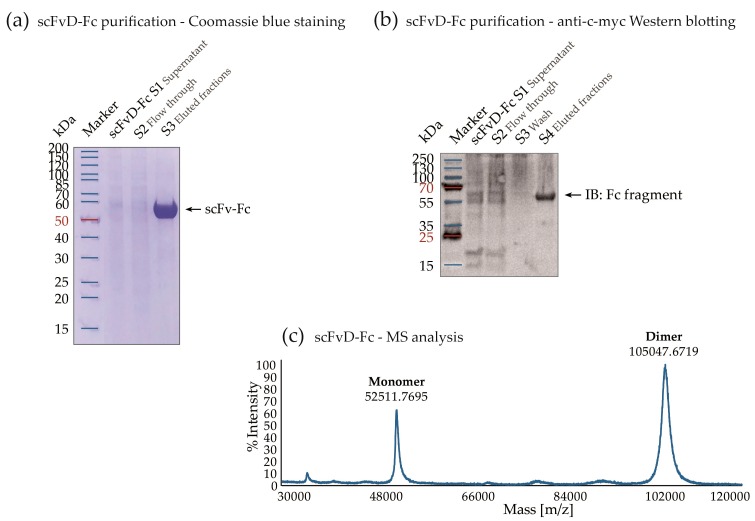
Purification and analysis of scFv-Fc fusions at an example of scFvD-Fc. (**a**) SDS-PAGE and Coomassie brilliant blue staining of the scFv-Fc purification steps; (**b**) Western blot analysis of the scFv-Fc purification process. The membrane was stained with anti-human IgG Fc fragment HRP-conjugated antibody; (**c**) The representative mass spectrum of purified scFv-Fc. The detected m/z peaks correspond to a covalent dimer and monomer of the scFv-Fc protein.

To increase the size and valency of the selected antibody fragments and therefore improve their avidity, we cloned scFvs A, C, and D into the scFv-Fc format. The Fc-fusions were designed by introducing the CH2 and CH3 domains of human IgG1 to the scFv constructs. scFv-Fc fusion proteins were expressed using transient transfection in CHO-S cells, resulting in moderate to high yields of recombinant protein expression (Table S2). Protein production was followed by a single-step affinity chromatography purification on Protein A resin with SDS-PAGE and Coomassie blue staining, anti-Fc Western blotting, and mass spectrometry analysis (Figure 7a–c). All scFvs-Fc showed the same purity level, estimated to be above 95%.

### 2.5. Kinetic Parameters of the scFv-Fc Antibody Fragments

**Figure 8:**
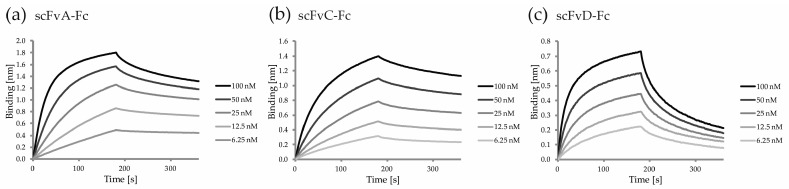
Sensograms for binding of serial two-fold dilutions of scFvs-Fc to FGF1. (**a**–**c**) Chemically immobilized FGF1 was used to capture analyzed scFvs-Fc, and the dissociation was measured in KB buffer.

As described in Section 2.3. for the scFvs, the affinity to FGF1 and the parameters of the selected scFv-Fc antibody fragments were measured with BLI and analyzed in the same manner. As expected, the bivalent antibody format demonstrated enhanced binding to FGF1, compared to the parental scFv, with binding affinities improved from 3- to 17-fold and reaching the nanomolar range (Figure 8a–c and Table 2).

### 2.6. Blocking of FGF1-dependent Cell Proliferation

**Figure 9:**
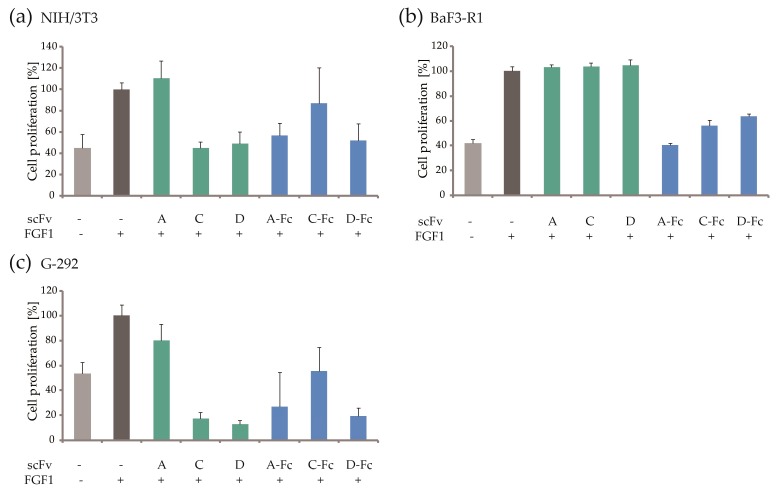
Antimitogenic FGF1 activity of selected antibody fragments tested on (**a**) NIH/3T3; (**b**) BaF3-R1; and (**c**) G-292 cell lines. The cells were starved for 24 h and incubated with scFvs (green) or scFvs-Fc (blue) in the presence of FGF1 and heparin, with cells without any treatment (light grey), and cells incubated with FGF1 and heparin only (dark grey), serving as controls. After 48 h of incubation, the cell proliferation level was determined. The error bars show standard deviation.

To explore the effects of generated antibody fragments on FGF1 mitogenic activity, FGF1-dependent cell proliferation was examined. The mouse fibroblast NIH/3T3 cells were treated with selected scFvs and scFvs-Fc without (Figure S1a) and in the presence of FGF1 and heparin (Figure 9a). By trapping FGF1, five selected antibody fragments—scFvs C and D, together with scFvs A-Fc, C-Fc, and D-Fc, efficiently inhibited NIH/3T3 cell proliferation, from 22 to 91%. Generated antibody fragments were further tested in FGFR1-expressing BaF3-R1 cells, in which scFvs-Fc also displayed significant activity (cell proliferation inhibition from 62 to 100%, as shown in Figure 9b), similar to the ECD_FGFR1-Fc ligand trap control (Figure S1b). The lack of effect for the scFvs may result from overall lower stability of this format in a long-term assay. This may also be the case for scFvA tested in NIH/3T3 cells, as it inhibits cell proliferation only as the Fc fusion. Finally, we also achieved a strong antimitogenic activity of the generated antibody fragments on G-292, FGFR1-positive, human osteosarcoma cancer cell line (Figure 9c), confirming their ability to efficiently bind FGF1 and preclude its binding to FGFR1 in in vitro model, without having any effect in the absence of FGF1 (Figure S1c). The results obtained for the cancer cell line were consistent with the data for fibroblast cells, showing the most significant effect for scFvs B and C, together with all generated scFvs-Fc. Similarly to NIH/3T3 cells, all antibody fragments except scFvA, showed complete proliferation inhibition.

## 3. Discussion

FGF1 is a strong mitogenic agent, important in angiogenesis, tissue regeneration, and the inflammation processes [8,27]. The role of FGF1 signaling in cancer is widely studied [6,19,28,29]. Preclinical and clinical evidences indicate that blocking aberrant FGF/FGFR signaling may represent a promising strategy for the therapy of human cancers [30]. Efforts targeting this pathway have led to the development of numerous small molecule FGFR inhibitors with wide implications for cancer therapy [9]. These tyrosine kinase inhibitors show varied effects in clinical trials, with the response being highly dependent on tumor genetic characteristics [31].

Another group of therapeutics consists of antibodies and FGF traps that represent a novel class of compounds with promising therapeutic features. Their advantages over the TKIs are low dosage and toxicity, high activity, and amino acids being their metabolic end products. There are several monoclonal antibody fragments currently being investigated in preclinical and early phase development studies [30,32]. The examples include: GP369, an antibody specific for FGFR2, or BAY1187982—an anti-FGFR2 antibody-drug conjugate, that both have shown successful results on gastric and breast cancer models [12,33]. Another drug, MFGR1877S, a monoclonal antibody targeting FGFR3 has demonstrated promising activity in preclinical models of urothelial carcinoma [15], although failed to work in clinical studies of multiple myeloma treatment.

Ligand traps, on the other hand, target soluble growth factors, and by sequestering them prevent activation of FGFRs on cancerous cells. This group is represented by natural FGFs binders, such as heparin, thrombospondin-1 (TSP-1), or longpentraxin-3 (PTX-3) [34], as well as soluble decoy FGFRs [17,35] and synthetic molecules able to bind FGFs, e.g., heparin-like polyanionic molecules [36] and anti-FGF1 antibodies [20,21,22]. To date, among the listed FGF traps, a promising molecule is FP-1039, a soluble FGFR1-Fc fusion protein that binds almost all FGFs, and thus inhibits growth of different tumor cell lines, including lung and endometrial cancer, as well as mesothelioma cell lines [35,37]. FP-1039 inhibits in vivo growth of different tumor models, shows low toxicity, and is currently tested in phase I clinical trial [16].

Among anti-FGF antibodies developed for therapeutic applications, FGF2 is the most commonly chosen target for selection of antibodies or antibody fragments [20,21,22], but there are also few reports on anti-FGF1 [24,38], anti-FGF8b [23], and anti-FGF23 [25]. Regarding antibodies binding FGF1, there is a scFv1C9 developed by the group of Xiao-Juan Zhu [24,38,39]. This small antibody is based on hybridoma derived from mouse immunized with FGF1 and inhibits growth of breast and glioma tumors in vitro and in vivo. Our experimental approach presented here for the identification of FGF1-binding scFv variants differs significantly, as they were selected from scFv library with the use of phage display. Additionally, the binders were counterselected against receptor-binding deficient FGF1 mutant to maximize the probability of selected scFv clones binding in the FGFR-FGF1 interface and thus interrupting receptor–ligand interaction.

In this study we developed a panel of antibody fragments capable of binding and neutralizing the activity of FGF1. We have selected this target, as the activity of FGF1 is related to the activation of all four FGF receptor isoforms [40], thus the potential of the generated antibody fragments can be more widely exploited. The binders were obtained by optimizing a well-established selection procedure from Tomlinson I and J libraries [41]. scFv—a widely used antibody format has several advantages over the full-length monoclonal antibodies, such as easier production, or improved penetration into tumor tissue [42]. Three scFv clones, named scFvA, C, and D, showing best binding properties, were selected for further experiments. First, we validated scFv binding to FGF1 by SAR-by-NMR measurements. The SAR-by-NMR method is based on monitoring the chemical shift changes in protein amide backbone resonances upon addition of an interacting agent [43,44,45]. For all tested scFvs we have observed changes in NH chemical shifts of ^15^N-FGF1, mostly by the broadening of the NMR signals and reduction of signal intensities due to complex formation, implying an intermediate chemical exchange rate on the NMR timescale [44,45,46]. Moreover, generated antibody fragments showed high selectivity against FGF1, with micro- and nanomolar dissociation constants, as established by BLI measurements.

In the next stage of our study we have reformatted the scFv fragments to the scFc-Fc fusion format. In line with our expectations and previous experience, this conversion resulted in significant improvement of the affinity to FGF1, lowering the dissociation constants by 3- to 17-fold. Moreover, when considering the future therapeutic applications, the scFv-Fc format displays several advantages, that include higher valency and larger size of the antibody fragments. It results in prolonged circulation half-life, bivalent binding, and possessing the ability to induce antibody-dependent cell-mediated cytotoxicity [47].

To explore the value of generated FGF1 traps as therapeutic agents, we determined their capacity to inhibit the FGF1-induced proliferation of FGFR-expressing cells. We performed cell proliferation assays on fibroblast NIH/3T3 cells, which are known to express FGFR1 and are the most responsive cells with regard to FGF1-induced mitogenic effects, and therefore optimal for proliferation assays. Additionally, we have tested BaF3-R1 pro B cells, overexpressing FGFR1 with Ig-like domain III in c splice form [48]. BaF3-FGFR1 cells respond well to FGF1 stimulation (similarly to NIH/3T3) and are therefore a good model of FGFR-overexpressing cancers for in vitro proliferation assays. In the next step we have examined the effect of selected antibody fragments on FGF1 trapping in human osteosarcoma G-292 cancer cell line. We demonstrated that all generated scFv-Fc fusions potently suppressed the FGF1-induced cell proliferation of all: fibroblast NIH/3T3 cells, transfected BaF3-R1 cells, and FGFR1-expressing G-292 cells. We did not observe antiproliferative activity for scFvA in the NIH/3T3 and G-292 models, and for the scFv fragments A, C, and D in the BaF3-R1 cells, presumably due to a possible lower stability of scFv format compared to Fc-fusions. Yet, two of the tested scFvs (C and D) have shown inhibitory effect on NIH/3T3, and G-292 proliferation.

In general, generated antibody fragments in Fc-fusion format effectively neutralized the mitogenic activity of FGF1, and the level of cell proliferation showed dramatic reduction up to 100% for the scFvA-Fc in the BaF3-R1 model, and for the majority of tested antibody fragments in the G-292 model.

Summarizing, with the use of an optimized phage display method and subsequent reformatting, we have generated highly potent anti-FGF1 antibody fragments, exhibiting high affinity and specificity towards the antigen. Our data clearly indicate that the developed antibody fragments acting as FGF1 ligand traps can serve as potent therapeutic agents for the treatment of FGF1-dependent cancers. Further studies on cancer cell lines continued in in vivo xenograft models will be essential to future development of generated anti-FGF1 molecules.

## 4. Materials and Methods

### 4.1. Proteins

#### 4.1.1. FGF1 and FGF1 Mutant Y94A/N95A

Recombinant FGF1 (Met-Ala-FGF1^22−155^) was produced at 37 °C in an *E. coli* BL21(DE3)pLysS strain and purified on a heparin-sepharose CL-6B column following a described procedure [49,50]. FGF1 mutant Y94A/N95A was designed by Zakrzewska et al. [26]. The recombinant protein was expressed and purified in the same manner as for FGF1.

#### 4.1.2. ^15^N FGF1

^15^N-FGF1 for NMR measurements was expressed in *E. coli* BL21(DE3)Rosetta grown in minimal medium containing ^15^N-NH_4_Cl as nitrogen source. 30 mL of preculture in LB medium was centrifuged, washed, and used to inoculate minimal medium M9 with ^15^N-NH_4_Cl and 0.4% glucose. Cells were grown at 37 °C to OD_600_ = 1.2 and induced with 0.5 mM IPTG. Protein expression was conducted for 16 h at 30 °C. Protein from soluble fraction was purified on a heparin-sepharose CL-6B column similarly to wild-type FGF1.

#### 4.1.3. FGF2

Recombinant, 133 amino acid truncated form of FGF2 was expressed at 25 °C in *E. coli* BL21(DE3)pLysS and purified by affinity chromatography as described by Swiderska et al. [51].

#### 4.1.4. ECD_FGFR1-Fc

Human IgG1 Fc fusion of the full length extracellular domain of FGFR1 (ECD_FGFR1-Fc) expression was carried out according to Sokolowska-Wedzina et al. [52] with minor modifications. Briefly, CHO-S cells (Invitrogen, Carlsbad, CA, USA) were cultured at 37 °C in a shaking incubator at 110 rpm with 8% CO_2_ in PowerCHO-2CD medium (Lonza, Basel, Switzerland) supplemented with 8 mM l-glutamine and 1× penicillin/streptomycin solution (Biowest, Riverside, MO, USA). On the day of transfection the culture was centrifuged and cell pellet was resuspended in ProCHO4 medium (Lonza). Appropriate amount of ECD_FGFR1-Fc encoding plasmid (1.25 μg of DNA per every 1 × 10^6^ cells) was resuspended in 150 mM NaCl, mixed and incubated for 10 min at RT with a proper amount of linear polyethylenimine (PEI; Polyscience, Warrington, PA, USA) and diluted in 150 mM NaCl (5 μg of PEI per each 1 × 10^6^ cells). The final volume of the DNA and PEI solution corresponded to 10% of the total cell suspension volume. The DNA and PEI complexes were added to the cell solution. The transfection procedure was carried out for 4 h at 37 °C, 110 rpm, and 8% CO_2_. After this time, the culture was diluted with an equal volume of PowerCHO-2CD supplemented with 8 mM l-glutamine and 2× penicillin-streptomycin solution and incubated at 32 °C, 110 rpm, and 8% CO_2_. On the second day of protein production, the CHO-S culture was supplemented with l-glutamine to the final concentration of 4 mM. The cells were harvested on the tenth day of expression. The purification of the ECD_FGFR1-Fc protein was performed following a described procedure, using affinity chromatography rProtein A Sepharose Fast Flow Resin (GE Healthcare, Little Chalfront, UK) [52].

### 4.2. Phage Display Selection of scFv Antibody Fragments

The selection procedure was based on a Tomlinson I and J libraries protocol [41] and was performed as follows. The 96-well Nunc Maxi Sorp plates (Thermo Fisher Scientific, Waltham, MA, USA) were coated overnight at 4° C with 100 μg/mL of FGF1 or FGF1 Y94A/N95A in PBS. After washing and blocking with 2% MPBS (Marvel milk in PBS) the counterselection wells coated with FGF1 Y94A/N95A were incubated with 10^12^ phage particles from the Tomlinson I and J libraries (Source BioScience, Nottingham, UK), blocked with 2% MPBS, for 40 min with rotation (600 rpm) and 80 min standing at 4 °C. The phage particles were then transferred to the washed and blocked selection wells coated with FGF1 and incubated as described for FGF1 Y94A/N95A. The wells were then washed 10 times with PBS-0.1% Tween 20 and 10 times with PBS (15 and 20 times for each buffer for the subsequent rounds). For panning rounds 1 and 2 the bound phage was eluted with 100 mM triethylamine (TEA) and neutralized with 1 M Tris-HCl, pH 7.2. In the third round the selected phage particles were unbound from the ligand for 3 h by elution with 2-times molar excess of ECD_FGFR1-Fc over the immobilized FGF1. The second elution step in this round was performed with TEA as in the previous rounds. Propagation of phage after each round of panning was carried out as described by Viti et al. [53] and Lee et al. [41]. The monoclonal ELISA of soluble scFv antibody fragments was carried out according to Sokolowska-Wedzina et al. [54].

### 4.3. Biolayer Interferometry Screening of scFv Antibody Fragments

Bacterial supernatants containing soluble scFvs (the same samples as used for ELISA) were filtered through 0.22 μm filters and screened for binding to FGF1 by biolayer interferometry. BLI experiments were performed on an Octet K2 instrument (ForteBio Inc., Menlo Park, CA, USA) using amine-reactive (AR2G) sensors. Sensor tips were hydrated for 15 min prior to use. The sensors were then activated with a freshly prepared mixture of 20 mM EDC and 10 mM sulfo-NHS, coupled with 20 μg/mL FGF1 in 10 mM sodium acetate, pH 6.0, and then excess reactive esteres were blocked with 1 M ethanolamine, pH 8.5. Amine-coupled FGF1 was then used to capture scFvs present in the filtered bacterial supernatants. Association and dissociation from the ligand were both monitored for 180 s each. Surfaces were regenerated with 100 mM glycine, pH 4.5, and the assay was repeated. ForteBio’s Data Analysis 9.0 software was used for the analysis of the binding curves.

### 4.4. Purification and Analysis of scFv Antibody Fragments

scFv antibody fragments were prepared according to Sokolowska-Wedzina et al. [54]. Briefly, purified pIT2 plasmids with scFv sequences were electroporated into *E. coli* HB2151 cells (Source BioScience). The bacteria were grown in 2× TY media supplemented with 100 μg/mL ampicillin and 0.1% glucose to OD_600_ = 0.8 and the production of protein was induced with 0.5 mM IPTG. Cells were cultured at 30 °C, 180 rpm, overnight and then the cultures were harvested, centrifuged twice at 4000 rcf, 4 °C for 40 min, and filtered using a Stericap PLUS bottle filter device (Merck Millipore, Darmstadt, Germany). scFv antibody fragments were purified from the supernatants by affinity chromatography using rProtein A Sepharose Fast Flow Resin (GE Healthcare), following the same protocol as described previously by our group for ECD_FGFR-Fc proteins [52]. Purified scFvs were analyzed by SDS-PAGE, Western blotting using anti-c-myc antibody, clone 9E10 (Santa Cruz Biotechnology Inc., Dallas, TX, USA), and mass spectrometry. The molecular masses of the proteins were verified by MALDI-TOF/TOF 4800 (Applied Biosystems, Foster City, CA, USA), using α-cyano-4-hydroxycinnamic acid as a matrix.

### 4.5. Nuclear Magnetic Resonance Measurements

NMR spectra were measured for the 200 µL samples of 0.2 mM free ^15^N FGF1, 0.1 mM free scFv, and 0.1–0.12 mM FGF1-scFv complex. FGF1 protein was in 25 mM Tris-HCl, pH 7.5, 2 M NaCl, and 1 mM EDTA, and scFvs were in PBS buffer. 10% (*v*/*v*) D_2_O was added to the samples to provide a lock signal. All spectra were recorded at 300 K using an Avance 600 MHz spectrometer (Bruker, Billerica, MA, USA). ^1^H−^15^N heteronuclear correlations were obtained using the SOFAST HMQC pulse sequence [55].

### 4.6. Cross-Reactivity Assay

The Nunc Maxi Sorp 96-well plates were coated overnight at 4 °C with 50 μg/mL of FGF1, FGF1 previously thermally denatured by heating to 98 °C for 5 min, or FGF2 in PBS buffer. Next, the wells were washed with PBS and blocked with 2% MPBS for 100 min and after second washing with PBS, 10 μg/mL of scFvs were added to the wells and incubated for 60 min at 4 °C. The unbound proteins were washed and anti-c-myc mouse monoclonal antibody, clone 9E10 (Abcam, Cambridge, UK) was added to wells and incubated for 60 min at RT. The wells were washed with PBS and a secondary goat anti-mouse IgG antibody conjugated with horseradish peroxidase (115-035-003, Jackson ImmunoResearch, West Grove, PA, USA) was used. ELISA was developed using TMB liquid substrate (Sigma-Aldrich, Saint Louis, MO, USA). The reaction was stopped with 1 M H_2_SO_4_ and the absorbance was measured at 450 and 650 nm. The obtained absorbance values were a result of A_650_ background subtraction from A_450_.

### 4.7. Biolayer Interferometry Studies of FGF1 Binding Affinity

The kinetics of binding of antibody fragments to FGF1 were measured on an Octet K2 instrument at 25 °C with FGF1 being immobilized on the AR2G sensor surface in the same manner as described in 4.3. Measurements were performed in the PBS-based Kinetics Buffer (KB; ForteBio Inc.), association and dissociation of the analyte to and from the ligand were monitored for 300 s or 180 s for scFvs and scFvs-Fc, respectively. Sensor surfaces were regenerated with 100 mM glycine, pH 4.0. All measurements were performed in duplicate. The kinetic data were fitted and analyzed with the BIAevaluation 4.1 software (GE Healthcare) using a Bivalent analyte binding model [56,57] and respective rate constants (k_on_ and k_off_) and K_D_ values were calculated.

### 4.8. Epitope Binning with Biolayer Interferometry

FGF1 was immobilized on AR2G sensors as described in 4.3. Measurements were performed in the Kinetics Buffer. Amine-coupled FGF1 was used to capture first scFv at 200 nM for 180 s and then the sensor was moved to the well with the second scFv prepared at the same concentration and the following association was measured for further 180 s. The surface was regenerated with 100 mM glycine, pH 3.5, and the assay was repeated for another scFv-scFv pair. The measurements were continued until all available scFv-scFv configurations were examined. For the scFv-FGFR1 epitope binning assay, ECD_FGFR1-Fc was coupled with activated AR2G sensors, as described for FGF1 in 4.3., at 5 μg/mL in 10 mM sodium acetate, pH 5.0. FGF1 at 1 μM was preincubated with scFv fragment at 1:1 molar ratio for 30 min, and then the ability of complexed FGF1 to bind to the immobilized ECD_FGFR1-Fc was assessed. The sensor surface was regenerated with 100 mM glycine, pH 2.5, and the assay was repeated for the remaining FGF1-scFv complexes. Binding of FGF1 only and unspecific binding of scFvs to the immobilized protein was also verified. All measurements were performed in duplicate. The binding curves were analyzed using ForteBio’s Data Analysis 9.0 software.

### 4.9. scFv-Fc Fusions Preparation, Purification and Analysis of the Proteins

The scFv-Fc constructs were prepared as described by Sokolowska-Wedzina et al. [54]. Briefly, sequences encoding secretion signal peptide (SSP) and the HindIII and Kpn2I restriction sites were introduced into the scFv DNA sequences in two PCR reactions. In the next step, amplified scFv DNA and pLEV113-Fc expression vector (LakePharma, Belmont, CA, USA), encoding the Fc domain of human IgG1 [52], were digested using HindIII and Kpn2I restriction enzymes (Thermo Fisher Scientific), and then the PCR product was ligated with the vector with the use of T4 DNA ligase (Thermo Fisher Scientific). The resulting construct, inserted into a pLEV113 expression vector, was used to stably transfect CHO-S cells. scFv-Fc fusion proteins preceded with a secretion signal peptide were expressed and purified using affinity chromatography following the procedure described for the ECD_FGFR-Fc proteins [52]. The proteins were analyzed by SDS–PAGE, Western blotting using anti-human IgG Fc-HRP-conjugated antibody (ab97225, Abcam) and mass spectrometry.

### 4.10. Cell Proliferation Studies

Mouse embryo fibroblast NIH/3T3 and human osteosarcoma G-292 cells were obtained from American Type Culture Collection (ATCC, Manassas, VA, USA), whereas the BaF3-R1 (isoform IIIc) cell line was provided by David M. Ornitz from Washington University (St. Louis, MO, USA). NIH/3T3 and G-292 cells were seeded in a 96-well flat-bottomed plates (VWR, Radnor, PA, USA) at a density of 1 × 10^4^ cells/well in Dulbecco’s modified Eagle’s medium (DMEM, Biowest) supplemented with 10% fetal bovine serum (FBS, Biowest) and 1× penicillin/streptomycin solution (Biowest), while BaF3-R1 cells were seeded in a 96-well flat-bottomed plates (VWR) at 3 × 10^4^ cells/well in RPMI 1640 medium (Gibco, Thermo Fisher Scientific) supplemented with 10% bovine calf serum (BCS; Sigma-Aldrich), 1× penicillin/streptomycin solution (Biowest), 50 μM β-mercaptoethanol (Sigma-Aldrich), 4 mM l-glutamine (Biowest), and 0.5 ng/mL recombinant murine interleukin 3 (IL-3, PeproTech, Rocky Hill, NJ, USA) and incubated at 37 °C in a humidified incubator with 5% CO_2_ for 16 h. The cells were then washed with PBS and starved for 24 h in 80 μL/well of the same medium without the addition of FBS for NIH/3T3 and G-292, or IL-3 for BaF3-R1, respectively. 20 μL of activation medium containing FGF1 and heparin (Sigma-Aldrich) was added to the wells to the final concentrations of 5 ng/mL and 10 U/mL, respectively. scFvs and scFvs-Fc in 50 μL/well were added to the wells to a final concentration of 100 μg/mL and cells were allowed to proliferate at 37 °C, 5% CO_2_ for 48 h. Cell viability was measured using AlamarBlue or PrestoBlue reagent (Thermo Fisher Scientific), according to the manufacturer’s directions. Fluorescence emission was measured using an EnVision Multilabel Reader (PerkinElmer, Waltham, MA, USA). Each sample dilution was prepared in triplicate and every experiment was repeated on three independent plates.

## Figures and Tables

**Table 1:** Kinetic constants of the generated anti-FGF1 scFvs.

Protein	K_D1_ [M]	k_a1_ [M^−1^s^−1^]	k_d1_ [s^−1^]	K_D2_ [M]	k_a2_ [M^−1^s^−1^]	k_d2_ [s^−1^]
scFvA	1.41 × 10^−6^	2.98 × 10^4^	8.32 × 10^−2^	3.32 × 10^−5^	1.35 × 10^1^	4.49 × 10^−4^
scFvC	9.21 × 10^−6^	9.60 × 10^3^	4.19 × 10^−2^	2.05 × 10^−6^	2.13 × 10^2^	4.36 × 10^−4^
scFvD	1.21 × 10^−6^	2.14 × 10^4^	2.58 × 10^−2^	1.35 × 10^−5^	6.93 × 10^1^	1.35 × 10^−5^

**Table 2:** Kinetic constants of the anti-FGF1 scFvs-Fc.

Protein	K_D1_ [M]	k_a1_ [M^−1^s^−1^]	k_d1_ [s^−1^]	K_D2_ [M]	k_a2_ [M^−1^s^−1^]	k_d2_ [s^−1^]
scFvA-Fc	4.24 × 10^−7^	8.81 × 10^4^	3.72 × 10^−2^	7.27 × 10^−6^	1.64 × 10^2^	1.19 × 10^−3^
scFvC-Fc	5.22 × 10^−7^	7.24 × 10^4^	3.78 × 10^−2^	8.81 × 10^−6^	8.12 × 10^1^	7.16 × 10^−4^
scFvD-Fc	3.46 × 10^−7^	1.32 × 10^5^	4.56 × 10^−2^	6.52 × 10^−5^	5.13 × 10^1^	3.34 × 10^−3^

## Supplementary Materials

Supplementary materials can be found at http://www.mdpi.com/1422-0067/19/9/2470/s1.

## Abbreviations

BLI	Biolayer interferometry
CDR	Complementarity determining region
CHO	Chinese hamster ovary
ECD	Extracellular domain
EGFR	Epidermal growth factor receptor
ELISA	Enzyme-linked immunosorbent assay
Erk1/2	Extracellular signal-regulated kinases 1/2
Fc	Fragment crystallizable
FGF1	Fibroblast growth factor 1
FGFR	Fibroblast growth factor receptor
HMQC	Heteronuclear multiple-quantum correlation
HRP	Horseradish peroxidase
IB	Immunoblotting
KB	Kinetics buffer
MALDI	Matrix-assisted laser desorption/ionization
MS	Mass spectrometry
NMR	Nuclear magnetic resonance
RT	Room temperature
RTK	Receptor tyrosine kinase
SAR	Structure-activity relationships
scFv	Single chain Fragment variable
SDS-PAGE	Sodium dodecyl sulfate polyacrylamide gel electrophoresis
TEA	Triethylamine
TKI	Tyrosine kinase inhibitor
VEGFR	Vascular endothelial growth factor receptor
