# Supplementary material for: Specific Antibody Fragment Ligand Traps Blocking FGF1 Activity
Source: Int J Mol Sci. 2018 Aug 21;19(9):2470. doi: 10.3390/ijms19092470 (PMC6163658; doi:10.3390/ijms19092470)
Supplement: Supplementary file 1 [file ijms-19-02470-s001.pdf]

Supplementary Materials

Specific Antibody Fragment Ligand Traps Blocking FGF1 Activity

Table S1. Yields of individual scFv preparations.

Protein Name	Protein Yield [mg Protein/L Culture]
scFvA	3.2
scFvC	6.8
scFvD	29.7

Table S2. Efficiency of individual scFv-Fc preparations.

Protein Name	Protein Yield [mg Protein/L Culture]
scFvA-Fc	10.5
scFvC-Fc	29.2
scFvD-Fc	6.6

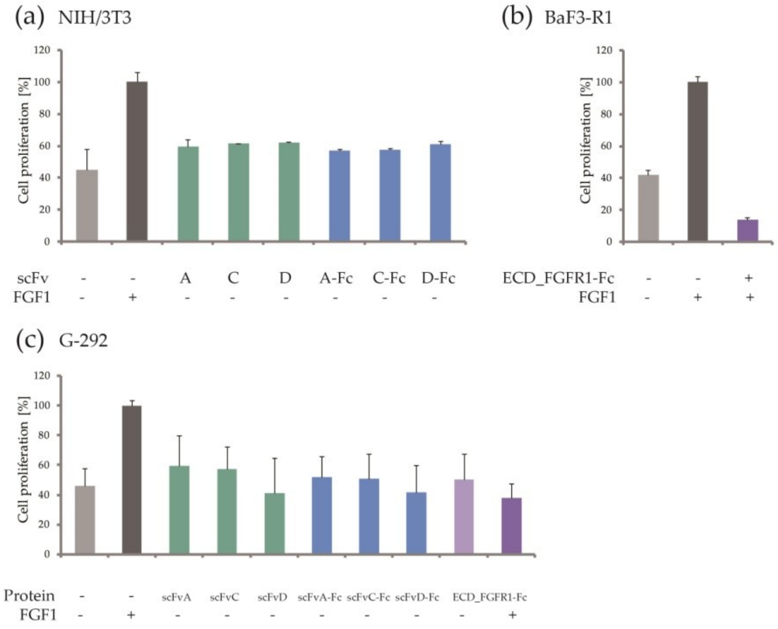

Figure S1. Influence on cell proliferation of selected antibody fragments and ECD_FGFR1-Fc. **(a)** NIH/3T3 cells were serum starved for 24 hours and incubated with scFvs (green) or scFvs-Fc (blue) in the absence of FGF1, with NIH/3T3 without any treatment (light grey), and NIH/3T3 incubated with FGF1 and heparin only (dark grey), serving as controls. After 48 hours of incubation, the cell proliferation level was determined. **(b)** BaF3-R1 cells were starved for 24 hours and incubated with ECD_FGFR1-Fc (dark purple) in the presence of FGF1, with BaF3-R1 without any treatment (light grey), and cells incubated with FGF1 and heparin only (dark grey), serving as controls. **(c)** G-292 cells were serum starved for 24 hours and incubated with scFvs (green), scFvs-Fc (blue), or ECD_FGFR1-Fc (light purple) in the absence of FGF1, with cells without any treatment (light grey), and G-292 incubated with FGF1 and ECD_FGFR1-Fc (dark purple), or FGF1 and heparin only (dark grey), serving as controls. The error bars show standard deviation.
